# Genetic variation associated with Marek’s disease resistance and susceptibility in white leghorn chickens

**DOI:** 10.1016/j.psj.2025.106311

**Published:** 2025-12-20

**Authors:** Christos Dadousis, Nicos Angelopoulos, Yaoyao Zhang, Anna Eleonora Karagianni, Huanmin Zhang, John A. Hammond, Venugopal Nair, Yongxiu Yao, Nophar Geifman

**Affiliations:** a School of Health Sciences, Faculty of Health and Medical Sciences, University of Surrey, Guildford, United Kingdom; b Veterinary Health Innovation Engine, School of Veterinary Medicine, Faculty of Health and Medical Sciences, University of Surrey, Guildford, United Kingdom; c The Pirbright Institute, Pirbright, Woking, United Kingdom; d School of Veterinary Medicine, University of Surrey, Guildford, United Kingdom; e The Roslin Institute and Royal (Dick) School of Veterinary Studies, University of Edinburgh, Easter Bush, Midlothian, United Kingdom; f USDA, Agricultural Research Service, US National Poultry Research Center, 934 College Station Road, Athens, GA, 30605, USA

**Keywords:** Chicken, Marek disease, Third generation sequencing, GWAS

## Abstract

Despite of effective control by vaccination, Marek’s disease virus (MDV) remains a significant threat to poultry health and productivity due to continued virus evolution, which drives the need to better understand host genetic factors underlying the resistance and susceptibility. Although great efforts have been made toward understanding the genetic resistance to MD, the genetic variations underlying varied susceptibility to MD remains poorly understood. In this study, we conducted a comprehensive genome-wide association and pathway enrichment analysis in chicken genomes of a MD-resistant inbred line 6 (LN6), a MD-susceptible inbred line 7 (SUS), and 6 recombinant congenic strains (RCS) derived from the lines 6 and 7. The incidence of MD in the RCS varied significantly, ranging from RCS-J – 0%, RCS-D – 4%, line 6 – 6%, RCS-F – 10%, RCS-W – 15%, RCS-K – 24%, to RCS-M – 41%. The progenital line 7 was observed with a 97% incidence in response to infection with a very virulent plus MDV strain (648A). Three models were constructed: *GWAS*_*LN6*_ with the line 6 birds contrasted against the remaining birds, *GWAS_SUS_* with the line 7 birds contrasted against the remaining birds, and *GWAS*_*RES-JD6*_ with group of RCS-J, RCS-D and line 6 together contrasted against the remaining birds. Our results revealed distinct enrichment patterns: while WNT/SHH Axonal Guidance Signaling Pathway is enriched in both *GWAS_SUS_* and *GWAS*_*RES-JD6*_, the Th2 pathway, Th1/Th2 Activation Pathway, and the interleukin (IL)-33 were predominant in *GWAS_SUS_*. On the other hand, the ISG15 antiviral mechanism and HIPPO signalling pathways were enriched in the *GWAS_RES-JD6_*. In contrast, thyroid cancer signalling, CXCR4, ILK, IL-8, IL-3, JAK/STAT and mTOR signalling pathways were significantly enriched in *GWAS_LN6_*. These findings underscore the complex interplay of immune signalling, host-pathogen interactions, and genetic regulation in shaping MD resistance. Key pathways and candidate genes identified in this study provide valuable targets for further functional validation and may inform future genetic selection and new vaccine strategies to enhance MD resistance in poultry.

## Introduction

Marek’s disease (MD) is a contagious, highly pathogenic, oncogenic and commercially important immunosuppressive and neoplastic disease in domestic (*Gallus gallus domesticus*) chickens. MD, first described by Josef Marek (Marek, 1907), impacts animal welfare and causes high economic losses in poultry industry worldwide (Morrow and Fehler, 2004a; Baigent et al., 2006; Berthault et al., 2018; Zhu et al., 2024). For example, the 2019 MD outbreak in Thailand caused ∼296 USD of total economic losses (Dejyong et al., 2023), while annual global losses have been estimated at ∼2 billion USD (Morrow and Fehler, 2004b).

MD is caused by *Gallid alphaherpesvirus* 2 (*Herpesviridae*; GaHV-2), widely known as the MD virus (MDV). It is considered to be a multifaceted disease with diverse physiological dysfunctions including immunosuppression (making chickens more susceptible to other diseases), lesions of the central and peripheral nervous system, and, diffuse tumours in the viscera. Apart from the direct symptoms, MD negatively affects production performance in poultry and increases morbidity (Zhu et al., 2024). Field diagnosis and disease control is difficult, with vaccination programmes often used to try and control MD. However, although vaccination prevents tumour development in most affected chickens, it doesn’t provide sterilizing immunity (controls disease rather eliminates MDV). Since the vaccinated chickens still support MDV replication and are potentially spreading MDV into the environment, it is considered as a source of infection to other susceptible chickens. Moreover, MD vaccine protective efficacy can drastically vary from line to line, with host genetics contributing to this variation (Xie et al., 2017). As a result, sporadic MD outbreaks emerge worldwide (Zhang et al., 2020). A reliable, economical, and robust alternative to control by vaccination is the leveraging of genetic resistance. MD genetic resistance is well established and one of the best examples for inherited resistance (Calnek, 1985; Vallejo et al., 1998). Two inbred lines have thus been developed, the highly resistant (RES; ∼100%) line 6 (LN6) and susceptible line 7 (SUS), to MD-induced tumours, in a breeding program that started in 1939 and completed in the 1970s. Later on the, the resistant line 6 and the susceptible line 7 were further crossed to produce F_2_ recombinants (Stone, 1975; Bacon et al., 2000). The development of the recombinant congenic strains (RCS) was proposed due to various phenotypic differences between LN6 and SUS that could be attributed to genetic correlations between traits and the polygenic and complex nature of susceptibility to diseases; this is common to animals and humans and poses difficulties in mapping genes associated with a specific trait. This includes the major histocompatibility complex (MHC) haplotype B*2 that affects MD resistance, yet is found in both the resistant line 6 and the susceptible line 7, indicating that genes located out of the MHC complex should affect MD resistance. To address this, the development of chicken RCS derived from the resistant line 6 and the susceptible line 7 was adopted (Bacon et al., 2000). The RCS approach was suggested as a tool for analysing polygenic traits, by partitioning a complex trait that has a polygenic genetic architecture into a series of single gene traits and was firstly developed in mice (Demant et al., 1989). Following this approach, each of the RCS have a random-unique ∼12.5% of the donor genome and ∼87.5% from the recurrent parent genome (Groot et al., 1992). Specifically for the development of the chicken MD-RCS the recurrent parent was decided to be the resistant line 6 and the donor was the susceptible line 7.

However, the development of vaccines in late 1960s has drastically decreased interest in breeding for genetic resistance, although genetic resistance could potentially be achieved in few generations (Cole, 1968). Interestingly enough, the first genetic studies on MD resistance reported higher vaccinal immunity and egg production of the resistant line compared to the susceptible chicken (von Krosigk et al., 1972; Spencer et al., 1974; Gavora and Spencer, 1979). Resistance to MD is reported at medium to high heritability of 61% (Gavora and Spencer, 1979), meaning that it can potentially be included in the breeding objective, and birds can be selected on MD resistance.

Previous mapping of quantitative trait loci (QTL) based either on a few hundreds of DNA markers in the form of microsatellites and linkage analysis, or based on single nucleotide polymorphisms (SNPs) and genome wide association analysis (GWAS), has found candidate genes related to MD resistance or susceptibility across the avian genome. More precisely, these were found on *Gallus gallus* autosomes (GGA) 1-7, 10-18, 24, 26-28, either by studying resistant and susceptible lines (Vallejo et al., 1998; Smith et al., 2020) or indigenous chicken breeds (Psifidi et al., 2016). More precisely, five QTL associated with MD susceptibility were on GGA 2,4,7,8 and on East Lansing genetic map (Cheng, 1997), 16 accounted for ∼23% of the phenotypic and ∼68% of the genetic variance (Vallejo et al., 1998). A more recent QTL analysis on MD resistance (Smith et al., 2020) detected 38 loci on GGA 1-7,10-14,16-18,24,26-28 and a list of 7 candidate genes on GGA1, 2 genes on each of GGA 13, 16 and 17 and from one candidate gene on GGA 2,4,14,18,24,26 and 27. Interestingly, the same study showed a sex differentiated regulatory mechanism to MD susceptibility, with genes involved in the Th1 immune response being more prominent in males, while genes in cancer signalling activation in females. Recently, genes and pathways related to MD resistance and/or susceptibility have also been reported in association with copy number variation (Bai et al., 2020) suggesting *IRF2* as a key gene related to MD susceptibility and resistance. Moreover, proteomic analysis in the spleen detected several pathways related to MD-induced apoptosis, inflammation and tumor, including Wnt, Hippo, TGF-B, P13K-Akt, mTOR, TNF, JAK-STAT, Calcium, NF-kB, MAPK, T and B cell receptor (Wang et al., 2024). More recently, heart tissue MD transcriptomic and metabolomic analysis in the Chinese Wenchang indigenous chicken showed that upregulated genes were related to immunity related pathways, while downregulated genes were involved in metabolic pathways (Xu et al., 2025), suggesting that chicken may supress cellular metabolism to counteract MD induced infection and tumor development.

Despite this, the genetic background of MD host resistance and susceptibility remains unclear, and genetic variations underlying varied susceptibility to MD in different lines of chickens remains poorly understood. A good understanding on the complete spectrum of genetic variation is crucial for elucidating disease mechanisms, as it provides comprehensive insights into the genetic factors contributing to diseases (Ebert et al., 2021). The third generation sequencing (TGS) is a promising tool for analysing complete individual genomes with reduced genotyping errors and increased read accuracy (Ross et al., 2013; Goodwin et al., 2016; Hufnagel et al., 2020). Moreover, TGS enables the detection of structural variants and epigenetic modifications, offering a more comprehensive understanding of individual genomes.

White Leghorn inbred lines of chickens, including the MD-resistant line 6, MD-susceptible line 7, and the RCS derived from the two highly inbred lines of chickens, have been extensively used for many years in efforts to investigate the genetic basis of disease resistance and susceptibility. By using the third generation PacBio whole genome sequencing of chicken genome on the selected birds from LN6, SUS, as well as the RCS with varying degree of resistance/susceptibility to MD (Xie et al., 2017), our objective was to elucidate genomic regions, genes, and biological pathways associated with MD resistance and susceptibility.

## Materials and methods

### Samples and genetic sequencing

A total of 16 birds were sequenced with the Pacific Bio-sciences (PacBio) TGS. Of these, two birds were from LN6, two birds were from the SUS line and 12 birds from the RCS (RCS- J, D, F, W, K and M with MD incidence of 0%, 4%, 10%, 15%, 24%, and 41% respectively), which were derived from the LN6 and SUS lines (MD incidence of 6% and 97% for line 6 and line 7, respectively) (Xie et al., 2017).

For each bird, structural variant calling was carried out in SMRT Link (12.0.0.177059) (Pacific Biosciences of California, Inc). Further, variant calling was conducted using DeepVariant −1.5.0 (Poplin et al., 2018). Biallelic SNPs with quality score >10 and variant depth > 10 were kept for subsequent analyses in BCFtools/1.17 (Li, 2011; Danecek et al., 2021). One bird from RCS-K was excluded due to low number of SNP (∼2.7 million). Following this step the dataset contained 15 birds with ∼4.5 million SNPs on *Gallus gallus* autosomes (GGA1 to 28), from which 2,588,954 SNPs were in common across all included birds. Median values for quality score were ∼64 across all birds and median variant depth ranged between 21 – 33 (Supplementary Table S1). Further quality control of the merged data applied in PLINK (v1.09) software (Purcell et al., 2007) (https://www.cog-genomics.org/plink/) included missingness per sample 10%, missingness per SNP 5%, and extreme deviation from Hardy-Weinberg equilibrium (*P* < 5 × 10^-5^). The chicken assembly bGal-Gal1.mat.broiler.GRCg7b (Ensembl release 108) was used as a reference genome (https://www.ncbi.nlm.nih.gov/datasets/genome/GCF_016699485.2/). Of the 2,588,954 SNPs, 2,558,309 (97.47%) had zero minor allele frequency in the dataset. Hence, 27,763 segregating SNPs were used for subsequent analysis. The SNP density maps of the ∼2.5 million and the ∼28k set of SNPs are presented in Supplementary Figure 1 (A and B, respectively).

### Principal component analysis

A principal component analysis (PCA) was conducted on the matrix of genotypes to visualise potential data stratification using the R (v. 4.4.1) function *prcomp* (R Core Team, 2021). The PCA applies a linear transformation of the genotypes into a set of mutual orthogonal vectors (principal components; PCs), where PCs are ordered by decreasing variability, and each of the PCs is a linear combination of all SNPs.

### Runs of homozygosity

Analysis of runs of homozygosity (ROH) was conducted in R (v. 4.4.1) (R Core Team, 2021) using the package *detectRUNS* (v. 0.9.5) (Marras et al., 2015; Biscarini et al., 2019). The consecutive runs method was used to define runs of homozygosity, with the length of 1Mbp and a minimum of 15 SNPs per ROH, and allowing for one heterozygous SNP within a ROH to account for potential genotyping errors. A ROH based inbreeding coefficient was estimated (F_ROH_). F_ROH_ refers to the proportion of the autosomal genome of an individual being in a ROH, and was estimated as the sum of ROH identified in an individual divided by the total genome length. F_ROH_ was summarised by chromosome for each group of birds.

### Linkage disequilibrium and average SNP distance

Genome-wide linkage disequilibrium (LD) was estimated as squared correlation based on genotypic allele counts in PLINK (v1.09) software (Purcell et al., 2007) (https://www.cog-genomics.org/plink/) using the flag --r2. Results were summarised per chromosome. Further, the average SNP distance per autosome was estimated.

### Genome-wide association analysis

Three GWAS were conducted, based on the binary phenotype comparisons: i) *GWAS*_*LN6*_, 2 line 6 birds (coded as 1) vs. all other birds (11 RCS and 2 line 7; coded as 0); ii) *GWAS*_*SUS*_, 2 line 7 birds (coded as 1) vs. all other birds (11 RCS and 2 line 6; coded as 0); iii) *GWAS*_*RES-JD6*_ (resistant birds including RCS-J, RCS-D and LN6), 2 birds of each LN6, RCS-J and RCS-D (coded as 1) vs. all other birds (RCS-F(2), -W(2), -K(1), -M(2) and 2 line 7; coded as 0).

In each of the three GWAS cases mentioned above, a single SNP regression was applied correcting for the background polygenic effect in GEMMA software (Zhou and Stephens, 2012):

y=1μ+wb+g+e,

where y is a binary vector; μ is the intercept, w is a column vector of genotypes for the SNP of interest with the corresponding allele substitution effect b; g is a vector of random additive genomic (polygenic) effects; and e is a vector of random residuals. The model assumptions were $g\;∼\;N\;(0,\;G_{\sigma_{g}}^{2})$ and $e\;∼\;N\;(0,\;I_{\sigma_{e}}^{2})$, where G is the centred genomic relationship matrix (flag *-gk 1*) and $\sigma_{g}^{2}$ and $\sigma_{e}^{2}$ are, respectively, the additive genomic and residual variances. A P-value threshold equal to 5 × 10−5 was adopted to declare significance (Burton et al., 2007). Annotation of all the significant SNPs was performed with the variant effect predictor (VEP; https://www.ensembl.org/Tools/VEP) program using the Ensembl database and the Galgal7 assembly.

### Gene-set enrichment and pathway-based analysis

Nominal P-values < 0.05 from the GWAS analyses were used to identify significant SNP. Using the *biomaRt* R package (Durinck et al., 2005, 2009) SNPs were assigned to genes if they were within the genomic sequence of the gene or within a flanking region of 15 kb up- and down- stream of the gene, to include SNPs located in regulatory regions. The size of the flanking region was based on the finding that most SNPs that affect the expression of a gene are located within 15 kb of the gene (Pickrell et al., 2010). For the assignment of the genes to functional categories, the Gene Ontology (GO) (Ashburner et al., 2000) was used. In addition, we ran the Database for Annotation, Visualization and Integrated Discovery web resource (Dennis et al., 2003) (DAVID) with the Kyoto Encyclopedia of Genes and Genomes (KEGG) pathway (Ogata et al., 1999) and UP_KW_Biological_Process, UP_KW_CELLULAR_COMPONENT, UP_KW_Molecular_Function and INTERPRO components. The background SNPs represent all the SNPs tested in the GWAS analyses, while the background genes were the genes associated with those SNPs. The GO database designates biological descriptors (GO terms) to genes based on attributes of their encoded products and it is further partitioned into 3 components: biological process, molecular function, and cellular component. The KEGG pathway database contains metabolic and regulatory pathways, representing the actual knowledge on molecular interactions and reaction networks.

The enrichment score (ES; a modified Fisher’s exact P-value test) calculated in DAVID was used to test for overrepresentation of the significant genes for each gene-set (i.e., pathway/ functional category), with higher ES reflecting more enriched clusters and ES > 1 means that the pathway/functional category is overrepresented.

Canonical pathways and networks of genomic regions associated with MD were identified using the Ingenuity Pathway Analysis (IPA) software (www.ingenuity.com). In brief, IPA constructs multiple possible upstream regulators, pathways and networks that serve as hypotheses for the biological mechanism underlying the phenotypes based on a large-scale causal network derived from the Ingenuity Knowledge Base. It then infers the most relevant pathways and networks by evaluating their statistical significance, with correction applied based on a baseline threshold (Krämer et al., 2014). The IPA score in the constructed networks was then used to rank these networks based on the P-values obtained using Fisher’s exact test [IPA score or P-score = –log10(P-value)].

## Results

### Susceptible chickens differ greatly from other lines of chickens

**Fig. 1:**
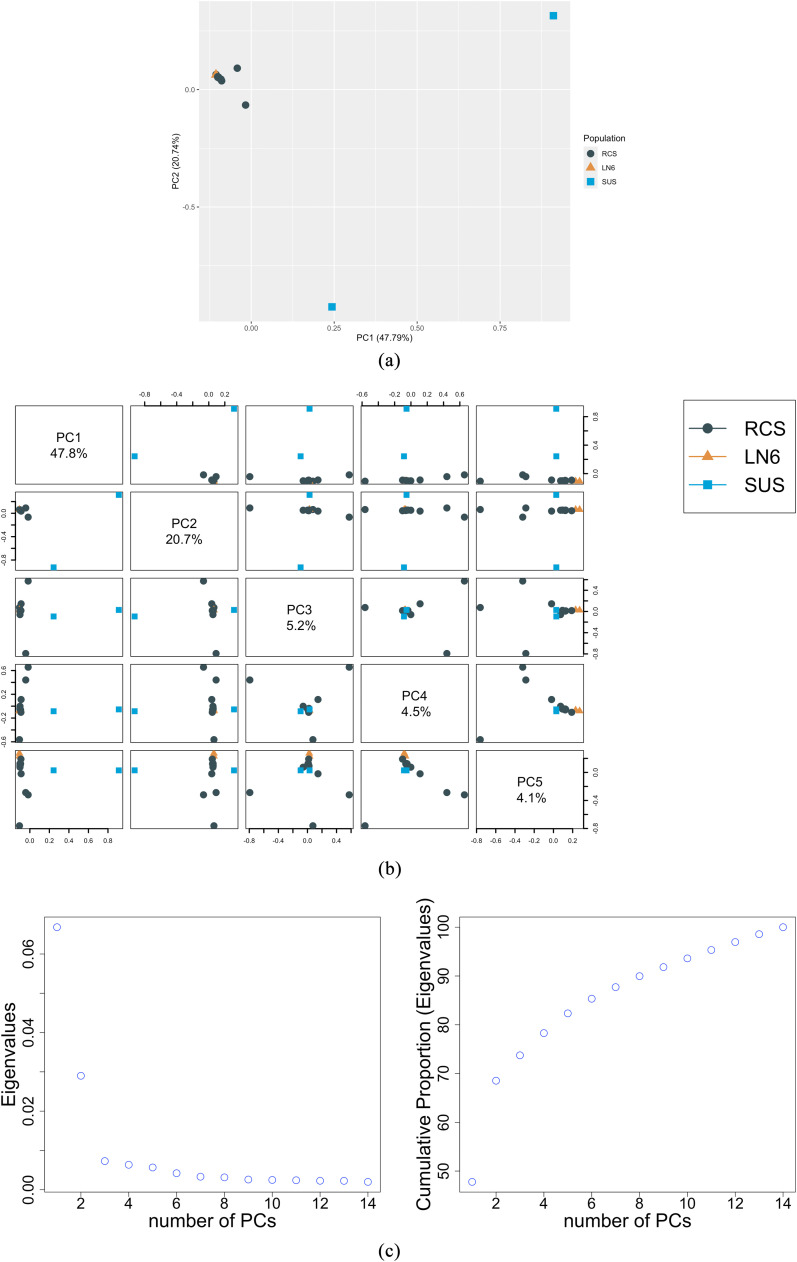
Results of the principal component analysis conducted on SNPs across the different genotypes: (a) Scatterplot and percentage of original variance explained of the first two principal components (PCs); (b) pairwise scatterplots of the first five PCs; and (c) variance and cumulative variance explained by the PCs. RCS: recombinants; LN6: resistant line 6; SUS: susceptible.

The PCA, presented in Fig. 1, shows that the two SUS birds of line 7 clearly separate from the RES and RCS birds. More precisely, the PC1, capturing 48% of the total variability, separates the SUS birds from the rest, while the PC2, capturing 21% of the variability, further separates the two SUS birds from each other, showing a considerable genomic variation between the two SUS birds. The PCs 3 to 4 (capturing cumulatively ∼9.7% of the variability) further split RCS in groups while PC5 slightly distinguishes the two LN6 birds from the RCS (Fig. 1B). The proportion of variance explained by each PC is summarized in Fig. 1C. Cumulatively, the first five PCs captured ∼82% of the original variability.

### Runs of homozygosity

**Fig. 2:**
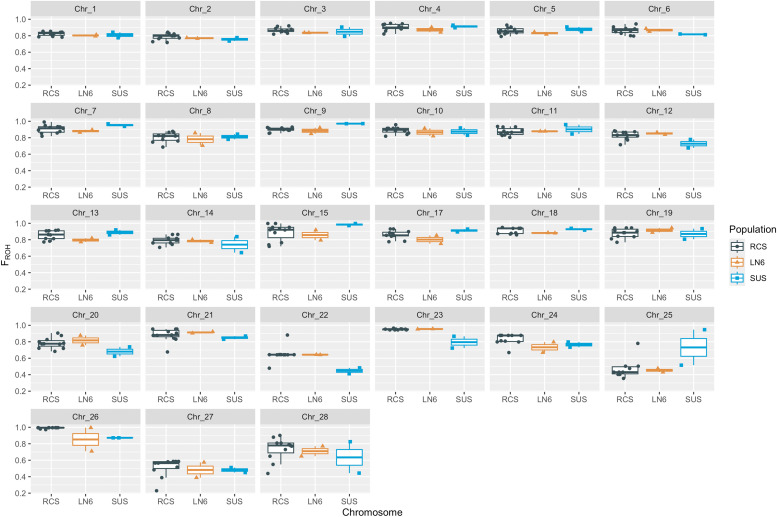
Summary of the genomic inbreeding coefficients based on runs of homozygosity per group of birds and chromosome. RCS: recombinants; LN6: resistant line 6; SUS: susceptible.

On average, we found 315, 322 and 306 ROH (average ROH length of ∼782, ∼763 and ∼772 Mbp), and an average F_ROH_ of 0.84, 0.82 and 0.83 for the RCS, LN6 and SUS, respectively. Variation among chromosomes was found (Fig. 2), with GGA22, 25 and 27 having the lowest F_ROH_ (<0.5 for all groups), with the exception of GGA25 for the SUS group where one bird had a F_ROH_ of ∼0.95. On GGA22, the LN6 birds had a F_ROH_ of 0.64, while the SUS birds of 0.48 and 0.41. The F_ROH_ for the RCS was 0.64 with the exception of D5014 and W2012 that had a F_ROH_ of 0.48 and 0.88, respectively. Conversely, on GGA25 the F_ROH_ of the LN6 birds was 0.43 and 0.48, while for the SUS birds 0.95 and 0.52. The lowest F_ROH_ on GGA25 was found for the RCS- M6372 (0.36) while F_ROH_ in the rest of the RCS varied between 0.40 – 0.50, with the exception of the RCS-D5014 where was found relatively high (0.78). On GGA27 F_ROH_ estimates varied between 0.23 – 0.59 but without a clear pattern.

### Linkage disequilibrium and average SNP distance

Average LD greatly varied among chromosomes with values ≥ 0.95 found on GGA 2, 3, 5, 6, 11, 15, 20, 22 and 23, and lowest (≤0.50) on GGA 17 and 26 (Supplementary Figure S2a). On the contrary, patterns of average SNP distance per chromosome was similar across GGA, with most of autosomes having ≤400 bp average SNP distance. An exception was found for GGA16 with ∼1.6 kbp average SNP distance, followed by GGA25 (∼800 bp average SNP distance).

### Genome-wide association analysis

**Fig. 3:**
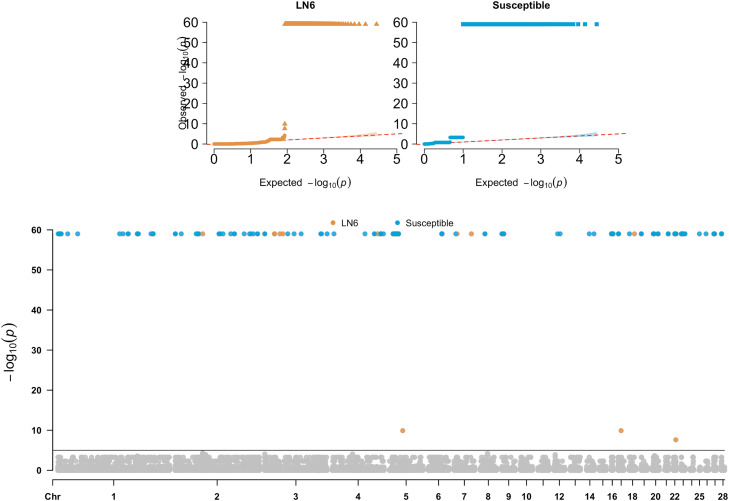
Manhattan plot of -log(P-values) and quantile–quantile (Q–Q) plots for the genome-wide association studies of the resistant and susceptible birds. Significance threshold was set to 5 × 10^-5^. P-values at zero were set at 10^-60^ for visualisation.
2) *GWAS_SUS_:* In the second GWAS analysis, the two SUS birds were contrasted against LN6 and RCS birds. In total, 2,750 significant associations were detected across the genome (GGA1-9, 12, 14, 16-23, and 25-28). Results of the susceptible GWAS are shown on Fig. 3 and Supplementary Table S2. In total, there were 1,248 significant associations found on GGA5 in five regions. From those, regions 5c (∼9.98 – 10.32 Mbp) and 5d (∼12.50 – 12.74 Mbp) included the majority of significant SNPs found on GGA5 for *GWAS*_*SUS*_ (639 and 597 SNPs, respectively; Supplementary Table S2); while significant SNPs on other positions on GGA5, namely ∼ 6.72 – 6.75 M; ∼ 14.31 – 14.57 Mbp; ∼ 16.40 – 16.46 Mbp, included 11 SNPs in total (1, 5 and 5 SNPs, respectively).

Three GWAS models were constructed with the aim of identifying SNP associations with MD (complete results available in Supplementary Table S2):

1) *GWAS*_*LN6*_: the two LN6 birds were contrasted against the SUS and RCS birds. In total, 326 significant SNPs were identified (P-value $\leq 5\;\times \;10^{-5}$) on GGA 1 (2; ∼1.16 and 112.3 Mbp), GGA 2 (2; ∼49.1 Mbp), GGA 3 (4; ∼17.2, 26.7 and 31.8 Mbp), GGA 4 (1; ∼79.86 Mbp), GGA 5 (310; ∼7.92– 8.23 Mbp), GGA 7 (2; ∼5.14 and 30.53 Mbp), GGA 17 (1; ∼5.0 Mbp), GGA 18 (1; ∼9.03 Mbp), GGA 22 (1; ∼ 4.11 Mbp), and GGA 23 (1; ∼62.28 Mbp). Manhattan and QQ-plots are presented in Fig. 3. The majority of significant associations (*n* = 310) were found on GGA 5 in a continuous region between ∼7.92– 8.23 Mbp.

Further, on GGA1 there were 569 significant SNPs in the region ∼167.23 – 167.47 Mbp. Apart from those, 14 regions were detected on GGA1, mainly with one or two significant SNPs, except the regions at ∼ 139.75 – 140.11 Mbp (15 significant SNPs), ∼2.19 – 2.20 and ∼3.37 – 3.39 Mbp (both with 6 significant SNPs), ∼4.61 – 4.65 Mbp (4 significant SNPs) and ∼2.41 – 2.45 Mbp (3 significant SNPs).

**Fig. 4:**
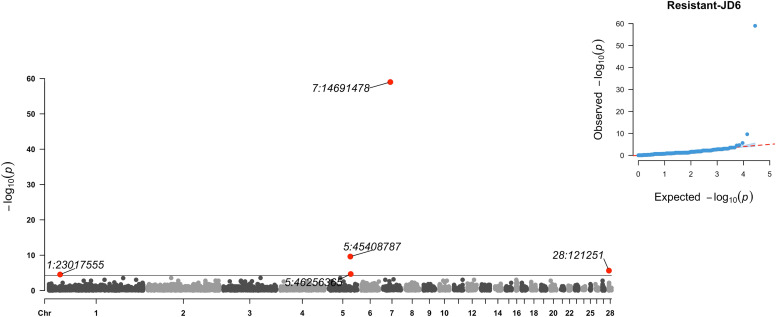
Manhattan plot of -log(P-values) and quantile–quantile (Q–Q) plot for the genome-wide association study of the resistant birds including the resistant recombinant lines J and D, and line 6. Significance threshold was set to 5 × 10^-5^. P-values at zero were set at 10^-60^ for visualisation.

3) *GWAS*_*RES-JD6*_ (resistant birds including RCS-J, RCS-D and LN6): in this study case, the four resistant recombinants from lines J and D were grouped together with the two line 6 birds and contrasted against the SUS and remaining RCS. In total, 5 significant SNPs were identified on GGA 1 (1), GGA 5 (2), GGA 7 (1), and GGA 28 (1). Manhattan and QQ-plots are presented in Fig. 4. The most significant SNP was on GGA 7 at ∼14.69 Mbp. On GGA5 the peak was at ∼ 45.41 Mbp and a second weak association at ∼ 46.26 Mbp. On GGA1 and GGA 28 two associations were identified at ∼23.02 Mb and ∼0.12 Mbp, respectively.

### Gene-set enrichment and pathway analysis

Out of a total of 27,763 SNPs, 989 (*GWAS*_*LN6*_), 10,800 (*GWAS*_*SUS*_) and 323 (*GWAS*_*RES-JD6*_) were located in annotated genes or in the 15 kb window (up-stream or down-stream from a gene). The total number of background genes annotated in the GalGal7 assembly was 6,115. From those genes, 181 were found in common between the *GWAS*_*LN6*_ and *GWAS*_*SUS*_, and 108 genes were found in common between *GWAS*_*RES-JD6*_ and *GWAS*_*SUS*_. For each of the lists of the genes a separate analysis was carried out, excluding the genes found in common. A flowchart of the gene-set enrichment analysis is presented in Supplementary Figure S3. The top GO and BP terms identified for each GWAS analysis are summarised in Table 1, while the full list in Supplementary Table S3.

**Table 1:** Gene Ontology (GO) Biological Process (BP) terms significantly enriched (P-value < 0.005) using genes associated with Marek’s disease.

GWAS	GO:ID^1^	Term^2^	P-Value	FDR
GWAS_LN6_	GO:0007275	multicellular organism development	2.0E-06	4.0E-04
	GO:0048856	anatomical structure development	4.8E-06	4.9E-04
	GO:0016043	cellular component organization	1.4E-05	9.6E-04
	GO:0048731	system development	3.4E-05	1.7E-03
	GO:0071840	cellular component organization or biogenesis	4.3E-05	1.7E-03
	GO:0036065	fucosylation	5.8E-05	1.7E-03
	GO:0009653	anatomical structure morphogenesis	5.8E-05	1.7E-03
	GO:0032501	multicellular organismal process	1.1E-04	2.9E-03
	GO:0032502	developmental process	1.8E-04	3.9E-03
	GO:2000045	regulation of G1/S transition of mitotic cell cycle	1.9E-04	3.9E-03
	GO:0030182	neuron differentiation	2.7E-04	4.8E-03
	GO:0048666	neuron development	2.8E-04	4.8E-03
	GO:0007017	microtubule-based process	3.6E-04	5.7E-03
	GO:0000902	cell morphogenesis	3.9E-04	5.7E-03
	GO:0048699	generation of neurons	4.5E-04	6.1E-03
	GO:0120036	plasma membrane bounded cell projection organization	5.5E-04	6.7E-03
	GO:1902806	regulation of cell cycle G1/S phase transition	5.8E-04	6.7E-03
	GO:0006996	organelle organization	5.8E-04	6.7E-03
	GO:0030030	cell projection organization	6.4E-04	6.8E-03
	GO:0007399	nervous system development	6.6E-04	6.8E-03
	GO:0000226	microtubule cytoskeleton organization	9.0E-04	8.9E-03
	GO:0031175	neuron projection development	1.0E-03	9.3E-03
	GO:0044707	single-multicellular organism process	1.0E-03	5.6E-01
	GO:0022008	neurogenesis	1.6E-03	1.4E-02
	GO:0036066	protein O-linked fucosylation	1.7E-03	1.4E-02
	GO:0007010	cytoskeleton organization	1.8E-03	1.5E-02
	GO:0044767	single-organism developmental process	2.2E-03	9.7E-01
	GO:0048812	neuron projection morphogenesis	2.5E-03	1.9E-02
	GO:0000082	G1/S transition of mitotic cell cycle	2.6E-03	1.9E-02
	GO:0120039	plasma membrane bounded cell projection morphogenesis	2.8E-03	1.9E-02
	GO:0048858	cell projection morphogenesis	2.8E-03	1.9E-02
	GO:0032989	cellular anatomical entity morphogenesis	2.8E-03	1.9E-02
	GO:0032436	positive regulation of proteasomal ubiquitin-dependent protein catabolic process	3.1E-03	2.0E-02
	GO:0051668	localization within membrane	3.3E-03	2.1E-02
	GO:2000060	positive regulation of ubiquitin-dependent protein catabolic process	3.5E-03	2.2E-02
	GO:0009101	glycoprotein biosynthetic process	3.6E-03	9.7E-01
	GO:0044843	cell cycle G1/S phase transition	4.6E-03	2.7E-02
	GO:0010256	endomembrane system organization	4.6E-03	2.7E-02
GWAS_SUS_	GO:0051090	regulation of DNA-binding transcription factor activity	1.0E-03	3.8E-02
	GO:0023058	adaptation of signaling pathway	1.2E-03	3.8E-02
	GO:0002029	desensitization of G protein-coupled receptor signaling pathway	1.2E-03	3.8E-02
	GO:0002031	G protein-coupled receptor internalization	1.2E-03	3.8E-02
	GO:0022401	negative adaptation of signaling pathway	1.2E-03	3.8E-02
	GO:0009987	cellular process	2.2E-03	4.2E-02
	GO:0006378	mRNA polyadenylation	3.0E-03	4.2E-02
	GO:0045744	negative regulation of G protein-coupled receptor signaling pathway	3.8E-03	4.2E-02
	GO:0006369	termination of RNA polymerase II transcription	3.8E-03	4.2E-02
GWAS_RES-JD6_	GO:0000226	microtubule cytoskeleton organization	3.2E-03	4.3E-02
	GO:0000422	autophagy of mitochondrion	3.8E-03	4.3E-02

1 GWAS_LN6_: 2 line 6 birds (coded as 1) vs. the rest (11 RCS and 2 SUS; coded as 0); GWAS_SUS_: 2 SUS (coded as 1) vs. the rest (11 RCS and 2 line 6; coded as 0); GWAS_RES-JD6_: 2 line 6 and 4 RCS (namely birds from lines J and D) that are resistant (coded as 1) vs. the rest (7 RCS and 2 SUS; coded as 0).

2 False discovery rate (FDR) correction for multiple testing.

Overall, 61, 40 and 9 significant (P-value < 0.005) GO terms were found associated with the genomic regions identified in *GWAS*_*LN6*_, *GWAS*_*SUS*_ and *GWAS*_*RES-JD6*_, respectively (Supplementary Table S3). From those, 38, 9 and 2 were BP terms. From those, “immunity” and “adaptive immunity” terms as well as “immunoglobulin domains” were enriched in the *GWA*_*SUS*_. Top in the enriched list were the terms CD8_asu, Adaptive immunity and Immunity, several terms related to immunoglobulin (Ig_V-set, Ig-like_dom_sf, Ig-like_dom, Ig-like_fold, Ig_sub, Ig-like, IGv and IG). Interestingly, the IPR015468:CD8_asu has also an overlapping homologous superfamily [Immunoglobulin-like fold (IPR013783)] in the InterPro database (https://www.ebi.ac.uk/interpro/entry/InterPro/IPR015468/).

**Fig. 5:**
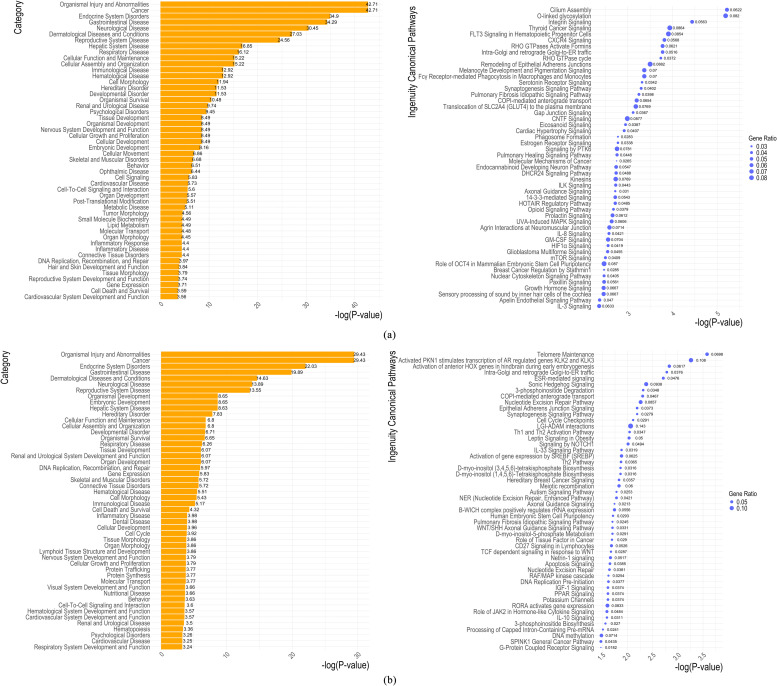

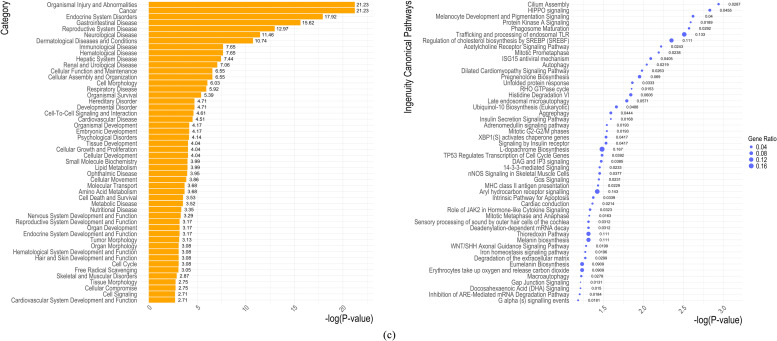
Top 50 disease and biofunctions (left) and canonical pathways (right) associated with Marek’s disease identified in IPA software: (a) *GWAS*_*LN6*_: 2 line 6 birds (coded as 1) vs. the rest (11 RCS and 2 SUS; coded as 0); (b) *GWAS*_*SUS*_: 2 SUS (coded as 1) vs. the rest (11 RCS and 2 line 6 birds; coded as 0); (c) *GWAS*_*RES-JD6*_: 2 line 6 birds and 4 RCS (lines J and D) birds that are resistant (coded as 1) vs. the rest (7 RCS and 2 SUS; coded as 0).

Several networks were constructed in IPA based on gene interactions from the set of genes identified in each GWAS analysis (Supplementary data). The top 50 disease and biofunctions, along with the top 50 canonical pathways associated with MD for each GWAS, are presented in Fig. 5. The number of canonical pathways shared across the three GWAS analyses is shown in Supplementary Figure S4. Five canonical pathways were shared between *GWAS*_*LN6*_ – *GWAS*_*SUS*_ (COPI-mediated anterograde transport, Synaptogenesis Signaling Pathway, Pulmonary Fibrosis Idiopathic Signaling Pathway, Intra-Golgi and retrograde Golgi-to-ER traffic, Axonal Guidance Signaling). Additionally, five pathways were shared between *GWAS*_*LN6*_ and *GWAS*_*RES-JD6*_ (Gap Junction Signaling, RHO GTPase cycle, Melanocyte Development and Pigmentation Signaling, 14-3-3-mediated Signaling, and Cilium Assembly)_;_ and two were in common between *GWAS*_*SUS*_ and *GWAS*_*RES-JD6*_ (Role of JAK2 in Hormone-like Cytokine Signaling and WNT/SHH Axonal Guidance Signaling Pathway). Of particular interest to MD can be the immunological disease that was shared across the three GWAS analyses, and the respiratory disease detected in the *GWAS*_*SUS*_ analysis. Moreover, enrichment was observed in the Th2 pathway, the Th1 and Th2 activation pathway, as well as the interleukin (IL)-33 and the Wnt/SHH axonal guidance signalling pathways in the *GWAS*_*SUS*_ analysis (Fig. 5b). In the *GWAS*_*RES-JD*6_, the interferon ISG15 (ubiquitin-like protein), Hippo signalling, and the Wnt/SHH axonal guidance signalling were enriched (Fig. 5c). In the *GWAS*_*LN6*,_ thyroid cancer signalling, as well as the CXCR4, ILK, IL-8, IL-3 and the JAK/STAT signallings, were statistically enriched.

**Fig. 6:**
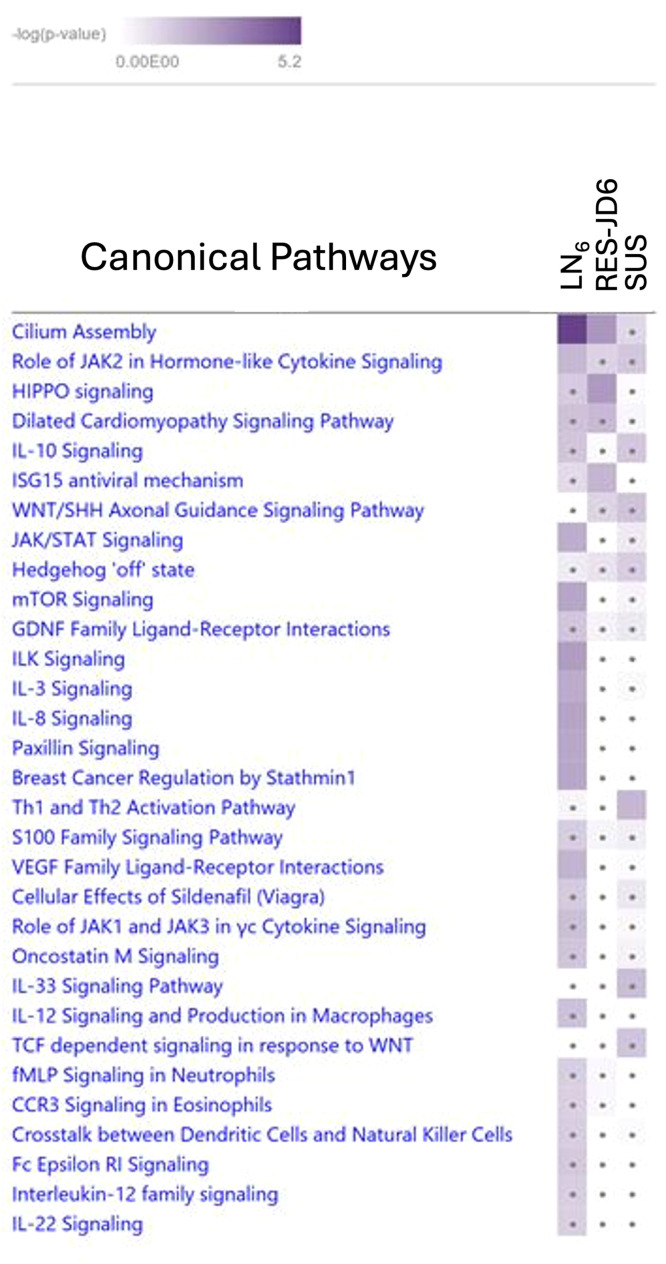
Comparison of canonical pathways identified in the three genome-wide association studies. Asterisk within cell implies non-significant for the given category. *GWAS*_*LN6*_: 2 line 6 birds (coded as 1) vs. the rest (11 RCS and 2 SUS; coded as 0). *GWAS*_*SUS*_: 2 SUS (coded as 1) vs. the rest (11 RCS and 2 line 6 birds; coded as 0). *GWAS*_*RES-JD6*_: 2 line 6 birds and 4 RCS (lines J and D) birds that are resistant (coded as 1) vs. the rest (7 RCS and 2 SUS; coded as 0).

A further comparison on the selected canonical pathways of immunological interest was carried out among the three canonical pathways identified in each of the three individual GWAS and pathway analyses. The results showed that the majority of the *GWAS*_*SUS*_ and *GWAS*_*RES-JD6*_ canonical pathways were not significant. In contrast, significant enrichment of the ILK, IL-8, IL-3, mTOR, Paxillin, and JAK/STAT signalling pathways was observed in *GWAS*_*LN6*_ (Fig. 6).

### Variant effect predictor

A VEP analysis was subsequently performed on the significant genes identified in the three genome-wide association studies. Overall, intronic variants were the most frequent (74, 53 and 70% for the *GWAS*_*LN6*_, *GWAS*_*SUS*_ and *GWAS*_*RES-JD6*_, respectively), followed by up/downstream gene variants (∼17% in all three cases) (Supplementary Figure S5). Four missense variants (two each for *GWAS*_*SUS*_ and *GWAS*_*RES-JD6*_) were found. More precisely, for the *GWAS*_*SUS*_ on GGA1 at 2,185,320 bp, the SNP rs739410388 was linked to G2/M phase-specific E3 ubiquitin-protein ligase-like gene (*LOC101750035*) and on GGA2 at 147,237,485 bp, the SNP rs734861409 is a missense variant of the lymphocyte antigen 6 complex, locus E (*LY6E*). Missense variants associated with resistance were detected on GGA1 at 115,701,282 bp (rs3555246657; ENSGALG00010003690 gene) and on GGA15 at 10,807,880 bp; (rs734317717; the SEC*14L3* gene, SEC14 like lipid binding 3) for *GWAS*_*RES-JD6*_.

## Discussion

Although there is considerable ongoing research on MD in chickens, the ways in which MDV affects and interacts with the host immune system remain largely unclear. In the current study, we aimed to identify genomic regions and pathways associated with MD in chickens, focusing on those modulating varying degrees of resistance and susceptibility. Despite the small number of birds analysed that could affect sample variation and thereby genomic similarity in our dataset, PCA results were as expected. In line with the RCS’ development that result in ∼87.5% of their genome coming from the resistant line 6 used as a recurrent parent (Groot et al., 1992; Bacon et al., 2000), the first two PCs (capturing 69% of the original variability) clearly separated the susceptible birds from the others, while also highlighting genomic heterogeneity within the susceptible birds. Similar grouping using multidimensional scaling on SNP genotypes from the Affymetrix Axiom® Genome-Wide Chicken Genotyping Array (600 K) was reported in Xu et al. (2018). High genomic inbreeding estimates were observed across all birds, ranging between 0.80 – 0.88. These estimates were expected since by 1952 the resistant line 6 and the susceptible line 7 were ∼95% inbred (Waters and Fontes, 1960) and the RCS are expected to be highly homogenous and are also considered inbred (Groot et al., 1992). Moreover, the high genomic inbreeding estimates were consistent with the findings reported by Xu et al. (Xu et al., 2018). By using whole genome third generation sequencing of 15 birds (resistant, susceptible, and recombinants) we carried out GWAS and pathway analyses. Three GWAS analyses were carried out, by contrasting i) resistant birds of the parental line 6 to all other birds (*GWAS*_*LN6*_), or ii) susceptible to all other birds (*GWAS*_*SUS*_), and finally iii) combined line 6 and recombinant resistant birds to the rest (*GWAS*_*RES-JD6*_). These comparisons resulted in the identification of complimentary genomic regions and pathways. In line with previous studies (Chakraborty et al., 2019), gene ontologies and KEGG pathways related to immunity were associated with susceptible birds, whereas genes linked to resistant birds were enriched for GO terms related to general cellular functions (Supplementary Table S3). Five canonical pathways were found in common between *GWAS*_*LN6*_ and *GWAS*_*RES-JD6*_, as well as between *GWAS*_*LN6*_ and *GWAS*_*SUS*_, while two pathways were common to both *GWAS*_*SUS*_ and *GWAS*_*RES-JD6*_ (Supplementary Figure S4).

### Interferon ISG15 and the JAK/STAT signalling pathway

ISG15 is considered to play a central role in host antiviral response (Perng and Lenschow, 2018). It also acts as a cytokine on lymphocytes (e.g., T and natural killer) to induce interferon (IFN)-γ production (Bogunovic et al., 2013). Interferons collectively play a crucial role in controlling and resisting pathogens by negatively regulate pathogen signalling through the JAK/STAT pathway (Schneider et al., 2014). On the other hand, viruses have evolved diverse countermeasures to evade or suppress ISG activity, allowing them to persist and replicate within the host. Evidence suggests that MDV activates the signal transducer and activator of transcription 3 (STAT3) to disables the ATR-Chk1 pathway, which is conducive to viral replication (Lian et al., 2019). Another *in vivo* study of MDV infection suggested that MDV downregulates mRNA and protein expression of IFN-α and IFN-β (Sun et al., 2019), while the interferon induced protein with tetratricopeptide repeats 5 (*IFIT5*) and the TNF alpha induced protein 2 (*TNFAIP2*) genes, that are affiliated within the interferon activation pathway, were increased after MDV infection in MD resistant birds (Warren et al., 2023). In addition, high level expression of signal transducer and activator of transcription 2 (*STAT2*) and signal transducer and activator of transcription 4 (*STAT4*) has been observed in MDV infected macrophages derived from MD susceptible chickens (line 7) compared to resistant chickens (line 6), suggesting dysregulation or overactivation of this pathway may correlate with disease progression (Chakraborty et al., 2019). The potential role of JAK/STAT signalling in response to MDV resistance has been highlighted in a recent study that analysed genome-wide copy number variations in MD-resistant chickens (Bai et al., 2020). Its involvement in response to MDV infection has been reported by transcriptome profiling of macrophage infection (Chakraborty et al., 2019), gene expression analysis by microarray hybridization (Smith et al., 2011), splenic proteome profiling following MDV infection in chickens (Wang et al., 2024), and using allele-specific expression and differential expression in broiler and layer chickens (Perumbakkam et al., 2013). Aligned with their roles in antiviral response, ISG15 was enriched in the *GWAS*_*RES-JD6*_ while the JAK/STAT signalling was enriched in the *GWAS*_*LN6*,_ further supporting for resistance-associated immune signalling.

### Interleukins in host defence mechanisms

Our analysis detected a set of interleukin signalling pathways enriched in *GWAS*_*SUS*_ (IL-33) and *GWAS*_*LN6*_ (ILK, IL-8, and IL-3). The disease outcomes of MDV are significantly influenced by the host's genetic makeup and immune competence (Xie et al., 2017). Interleukins (ILs) are pivotal cytokines that orchestrate immune responses during viral infections including those caused by MDV in chickens. Various interleukins have been implicated in the host's defence against MDV, either by promoting antiviral immunity or contributing to immunopathology and tumor development. For instance, IL-6 and IL-18 act through distinct pathways and may shape MD outcomes differently across tissues. IL-6 plays key roles in inflammation, immune response, and other physiological processes such as metabolism and hematopoiesis (Korn and Hiltensperger, 2021; Speake et al., 2022). Sustained IL-6 signaling is often linked to pro-tumorigenic and immunosuppressive milieus (Orange et al., 2023). IL-18 is a powerful pro-inflammatory cytokine that promotes Th1 activation primarily in macrophages and dendritic cells, often in synergy with IL-12. This activation results in the production of high levels of interferon-gamma (IFN-γ), which is critical for host defense against intracellular pathogens (Kaplanski, 2018; Ihim et al., 2022). Thus, the higher IL-6/IL-18 expression reported in splenocytes (Kaiser et al., 2003) and caecal tonsils (Heidari et al., 2014) of susceptible birds may reflect a dysregulated inflammatory state, whereas the reduced IL-18 seen in susceptible bone-marrow–derived macrophages (Chakraborty et al., 2019) suggests cell-type specific suppression or timing effects during MDV infection. Together, these data imply that susceptibility may involve excess IL-6–driven inflammation alongside insufficient or mis-timed IL-18–mediated Th1 activation in key myeloid compartments. Additionally, MDV encodes a viral interleukin-8 (vIL-8) homolog, which promotes lymphoma formation by attracting target immune cells (Parcells et al., 2001).

In our IPA analysis we observed that the IL-33 signalling pathway was significantly enriched in the *GWAS*_*SUS*_ (genes in pathway: *BCL2L1, H2AC20, H2BC17, IL1RN, MAP3K8*, and *PRKAR1A*), suggesting a potential role in disease progression. IL-33 functions as an alarm signal that is released upon tissue or cellular damage and is known to initiate and amplify type 2 immunity and inflammation. While direct evidence linking IL-33 to MDV pathogenesis is limited, its enrichment in susceptible birds may reflect excessive or dysregulated immune activation, potentially contributing to immunopathology or facilitating viral persistence during MDV infection. In a recent study of pancreatic cancer, IL-33 has been reported to activate ILC2s that further induce tertiary lymphoid structures (lymphoid aggregates that in chronically inflamed tissues, including cancer, infection and inflammation) (Amisaki et al., 2025).

Conversely, resistant line 6 chickens exhibited enrichment in several other interleukin-related pathways, including ILK (Integrin-Linked Kinase), IL-8, and IL-3 signalling pathways. These pathways are associated with immune cell recruitment (IL-8) (David et al., 2016), hematopoiesis and survival (IL-3) (Feng et al., 2022), and cell adhesion and signalling (ILK), all of which may support more effective immune responses and tissue integrity, contributing to more effective viral clearance and immune resilience. In particular, IL-8 signalling plays a direct role in MDV pathogenesis, as the virus encodes a viral homolog of IL-8 (vIL-8), which helps attract target immune cells. The distinct activation of these pathways in resistant line 6 birds suggests that a more balanced and coordinated immune response may contribute to resistance against MDV-induced disease.

### WNT signalling and other tumour related pathways

WNT and SHH signaling are classically involved in embryonic development and axon guidance but have also been implicated in oncogenesis and immune regulation (Katoh, 2005; Jiang and Hui, 2008; Kumar et al., 2021). In the context of MDV, dysregulation of developmental pathways like WNT and SHH may contribute to both tumorigenesis and virus-mediated neuropathology (Calnek, 2001). Our analysis revealed that the WNT/SHH Axonal Guidance Signaling Pathway was highly represented in both the susceptible (*GWAS*_*SUS*_) and resistant (*GWAS*_*RES-JD6*_) chicken groups. This dual enrichment suggests a complex role in MDV pathogenesis, potentially modulating both viral dissemination and host tissue repair or immune activation. Consistent with this, we have previously reported significant activation of the WNT signaling pathway during very virulent RB1B strain infection but not vaccine strain CVI988, both *in vitro* and *in vivo*, particularly noting its regulation in immune-related tissues and its interaction with host genes implicated in virus-host dynamics and tumor development (Xu et al., 2024). Furthermore, a splenic proteomic analysis by Wang et al. (Wang et al., 2024) in chickens infected with the MDV GX0101 strain confirmed activation of the general WNT signaling pathway, indicating that WNT-associated proteins are dynamically modulated during active infection. These converging lines of evidence underscore WNT signaling as a central mediator in both viral pathogenesis and the host’s immune-tumor interface, potentially influencing disease outcome across different host genetic backgrounds.

Interestingly, the Th2 pathway and the Th1 and Th2 Activation Pathway were specifically enriched in *GWAS*_*SUS*_ analysis. ​ An enrichment of Th2-related pathways in the susceptible birds could indicate an ineffective immune polarization against MDV, favouring a non-protective humoral response over a cytotoxic Th1-driven defence. This is in agreement with the finding that MDV induces Th2 activity during cytolytic infection (Heidari et al., 2008). The observation that a skewed Th2 response induced by pp38 peptides in an antigen specific manner in the MD-resistant chickens compared to that in the MD-susceptible chicken demonstrated that MDV infection impairs degranulation of T cells regardless of their genetic background (Boodhoo and Behboudi, 2022). The enrichment of Th1 and Th2 Activation Pathway in *GWAS*_*SUS*_ analysis may reflect an imbalanced or inadequate helper T cell response, especially in the absence of robust CD8⁺ T cell or innate responses. This supports the view that resistance to MD is likely multifactorial, involving not only adaptive immunity but also innate signaling networks and host regulatory pathways.

Conversely, the enrichment of the HIPPO signaling pathway from *GWAS*_*RES-JD6*_ analysis and pathways including the thyroid cancer signaling, CXCR4 signaling, and mTOR signaling pathways from *GWAS*_*LN6*_ analysis points to their potential roles in restricting MDV-induced tumorigenesis and enhancing antiviral immunity. The HIPPO pathway, which controls organ size and limits uncontrolled proliferation via YAP/TAZ transcription factors, is recognized for its tumor suppressive properties and has emerging roles in immune signaling (Yu et al., 2015). Likewise, the mTOR signaling pathway is a central modulator of cell metabolism, proliferation, and T-cell activation, and its balanced activity is critical for effective antiviral defence (Powell et al., 2012). In our study, both pathways were prominently enriched in resistant line 6 chickens, suggesting their involvement in maintaining cellular homeostasis and enhancing immune readiness against MDV. In support of this, Wang et al. identified significant alterations in both HIPPO and mTOR signaling pathways in the splenic proteome of chickens infected with the MDV GX0101 strain (Wang et al., 2024). These changes highlight active engagement of these pathways during infection, reinforcing their likely contribution to the host’s protective responses and tumor suppression during MDV challenge. Together, this aligns with our GWAS findings and underscores the importance of HIPPO and mTOR signaling in shaping resistance to MDV-induced oncogenesis.

Additional support for the relevance of the Wnt and mTOR signaling pathways in host response to MDV comes from a study on line 6 and line 7 chickens vaccinated with HVT and CVI988/Rispens, which identified differentially expressed miRNAs in the bursa that are predicted to regulate components of the Wnt and mTOR signaling pathways (Zhang et al., 2020). While this study did not confirm direct pathway activation, it implies that vaccine-induced modulation of host miRNAs may influence signaling cascades relevant to MDV resistance, further emphasizing the broader role of these pathways in host-virus interactions.

Moreover, of importance can be the four missense variants detected via post-GWAS VEP analysis in *GWAS*_*SUS*_ and *GWAS*_*RES-JD6*_. From those, the lymphocyte antigen 6 complex, locus E (*LY6E*) located on GGA2 at ∼147.2 Mbp has been previously suggested as a candidate MD resistant gene (Liu et al., 2003). More precisely, *LY6E,* that is involved in immune response via T cell activation and differentiation, was found to interact with the MDV protein US10. Although the LY6E gene has been previously suggested as a resistant gene, in our analysis it was detected only in the *GWAS*_*SUS*,_ indicating possible context-dependent roles or differences in strain/pathogen interaction. The other missense variant detected from *GWAS*_*SUS*_, the *LOC101750035* gene on GGA1 at ∼1.20 Mbp has been recently included in the top10 list (out of 3,320 upregulated and 1,264 downregulated genes) of significantly up-methylated N6-methyladenosine (m6A) genes in a study investigating the transcriptome m6A profiles of macrophages during Newcastle disease virus in chicken (Li et al., 2022), suggesting a potential regulatory role in viral immunity that warrants further investigation in MDV contexts. Selection of inbred lines of chickens was initiated for the identification of genes influencing resistance to avian tumours induced by oncogenic viruses. While line 6 and 7 birds showed genetic resistance and susceptibility respectively to MD, there were differences in the susceptibility to infection by different subgroups of avian leukosis viruses based on the presence or absence of specific virus entry receptors (Bacon et al., 2000).

Overall, using a series of complementary GWAS and post-GWAS analyses on chicken genome sequences and juxtaposing resistant with susceptible to Marek’s disease we were able to show that the genetic and immunological background of resistance/susceptibility differs in chickens with varying degrees of MD incidence. Even though our analysis utilized high coverage WGS of clearly distinct resistant and susceptible chicken and detected many significant SNP, gene ontologies and canonical pathways associated with MD, the limited number of samples may affect statistical power and biological interpretation, thus these results should be interpreted with caution. Further studies that will analyze large samples of high-coverage WGS or a mixture of SNP array genotyped and high-coverage WGS are needed to validate our results.

## CONCLUSION

To the best of our knowledge this is the first study on Marek’s disease analysing the complete genomes of resistant, susceptible, and recombinant birds using the third-generation sequencing. Our results suggest a polygenicity of the resistance and susceptibility of MD. Genes and pathways identified in our study could be used in the future for *in vitro* as well as *in vivo* studies targeting specific genes related to MD resistance and/or susceptibility. Our results also illustrate the usefulness of third generation genome sequencing and phenotyping set up in a small sample size, but well organised experimental design of inbred lines, for GWAS analysis in disease studies.

## CRediT authorship contribution statement

**Christos Dadousis:** Writing – original draft, Visualization, Software, Methodology, Formal analysis. **Nicos Angelopoulos:** Writing – review & editing, Visualization, Software, Methodology, Formal analysis. **Yaoyao Zhang:** Writing – review & editing, Data curation. **Anna Eleonora Karagianni:** Writing – review & editing, Visualization, Software, Methodology, Formal analysis. **Huanmin Zhang:** Writing – review & editing, Data curation. **John A. Hammond:** Writing – review & editing, Funding acquisition. **Venugopal Nair:** Writing – review & editing, Methodology, Investigation, Funding acquisition. **Yongxiu Yao:** Writing – review & editing, Supervision, Project administration, Investigation, Funding acquisition, Data curation, Conceptualization. **Nophar Geifman:** Writing – review & editing, Supervision, Project administration, Methodology, Investigation, Conceptualization.

## Disclosures

The authors declare that they have no competing interests.

## Appendix Supplementary materials

Image, application 1

Image, application 2

Image, application 3

Image, application 4

Image, application 5

Image, application 6

Image, application 7

Image, application 8
