# Supplementary material for: Genetic variation associated with Marek’s disease resistance and susceptibility in white leghorn chickens
Source: Poult Sci. 2025 Dec 20;105(3):106311. doi: 10.1016/j.psj.2025.106311 (PMC12819034; doi:10.1016/j.psj.2025.106311)
Supplement: Supplementary file 1 [file mmc1.docx]

**Supplementary Tables**

**Supplementary Table S1**: Measures of quality of whole genome sequencing per sample.

| Bird ID | No. of SNP after WGS QC^1^ | No. of SNP after excluding multi-allelic SNP | QUAL* | DP * |
| --- | --- | --- | --- | --- |
| J5339 | 4,553,469 | 4,545,998 | 64.30_8.84_ | 27.00_8.77_ |
| J5340 | 4,520,559 | 4,512,721 | 64.60_8.73_ | 31.00_10.07_ |
| D5019 | 4,605,670 | 4,598,244 | 64.30_8.88_ | 28.00_8.99_ |
| D5014 | 4,668,611 | 4,661,528 | 64.20_8.97_ | 28.00_9.02_ |
| 63R793 | 4,480,838 | 4,472,955 | 64.40_8.67_ | 28.00_9.25_ |
| 63Q941 | 4,419,151 | 4,411,400 | 64.10_8.47_ | 25.49_7.79_ |
| F5781 | 4,534,777 | 4,527,670 | 63.10_8.76_ | 21.00_7.21_ |
| F5815 | 4,507,223 | 4,499,686 | 64.10_8.64_ | 25.00_7.95_ |
| W2015 | 4,523,823 | 4,515,919 | 64.80_8.84_ | 33.00_10.94_ |
| W2012 | 4,518,101 | 4,510,243 | 64.70_8.79_ | 31.00_10.30_ |
| K5360 | 4,486,342 | 4,478,524 | 64.50_8.74_ | 26.00_8.65_ |
| M6372 | 4,552,505 | 4,544,933 | 64.60_8.86_ | 28.00_9.21_ |
| M6382 | 4,529,246 | 4,521,731 | 64.50_8.78_ | 28.00_9.18_ |
| 72Q1199 | 4,441,883 | 4,434,153 | 64.30_8.55_ | 25.00_8.01_ |
| 72R1309 | 4,513,771 | 4,505,894 | 64.40_8.79_ | 27.00_9.44_ |

^1^Whole genome sequencing (WGS) quality control (QC) included: autosomes 1-28, quality score (QUAL) >10 and variant depth (DP) > 10.

^*^Median and SD (supscript) values of QUAL and DP per sample.

**Supplementary Table S2:** Summary of the genome-wide associations (P-value < 0.00005).

| **GGA**^1^ | **No. of SNPs (signals)**^2^ | **Interval (Mbp)**^3^ | **P-value (range)**^4^ | **Top SNP location or range (bp)**^5^ | **GWAS**^6^ |
| --- | --- | --- | --- | --- | --- |
| 1a | 1 | 0.43 | 0 | 433728 | GWAS_SUS_ |
| 1b | 1 | 1.16 | 0 |  | GWAS_LN6_ |
| 1c | 6 | 2.19 – 2.20 | 0 | 2,185,320- 2,201,352 | GWAS_SUS_ |
| 1d | 3 | 2.41 – 2.45 | 0 | 2,411,827- 2,454,802 | GWAS_SUS_ |
| 1e | 6 | 3.37 – 3.39 | 0 | 3,367,974-3,389,569 | GWAS_SUS_ |
| 1f | 4 | 4.61 – 4.65 | 0 | 4,608,281-4,649,656 | GWAS_SUS_ |
| 1g | 1 | 5.90 | 0 | 5,897,220 | GWAS_SUS_ |
| 1h | 1 | 17.14 | 0 | 17,146,028 | GWAS_SUS_ |
| 1j | 1 | 23.02 | 3 x 10^-5^ | 23,017,555 | GWAS_RES-JD6_ |
| 1k | 1 | 34.75 | 0 | 34,748,519 | GWAS_SUS_ |
| 1l |  | 108.58 | 0 | 108,583,833 | GWAS_SUS_ |
| 1m | 1 | 112.87 | 0 | 112,886,721 | GWAS_LN6_ |
| 1n | 1 | 114.43 | 0 | 114,431,239 | GWAS_SUS_ |
| 1o | 2 | 123.13 – 123.90 | 0 | 123,125,055-123,904,655 | GWAS_SUS_ |
| 1p | 15 | 139.75 – 140.11 | 0 | 139,746,514-140,110,242 | GWAS_SUS_ |
| 1q | 1 | 141.71 | 0 | 141,712,441 | GWAS_SUS_ |
| 1r | 1 | 163.71 | 0 | 163,707,631 | GWAS_SUS_ |
| 1s | 569 | 167.23 – 167.47 | 0 | 167,228,760-167,470,214 | GWAS_SUS_ |
| 2a | 3 | 1.00-1.01 | 0 | 1,003,903-1,010,640 | GWAS_SUS_ |
| 2b | 1 | 11.42 | 0 | 11,420,100 | GWAS_SUS_ |
| 2c | 1 | 37,60 | 0 | 37,600,545 | GWAS_SUS_ |
| 2d | 46 | 40,54-40.57 | 0 | 40,542,913-40,571,748 | GWAS_SUS_ |
| 2e | 1 | 42.07 | 0 | 42,071,513 | GWAS_SUS_ |
| 2f | 2 | 49.06 – 49.07 | 4.79 x 10^-5^ – 0.00 | 49,060,987 | GWAS_LN6_ |
| 2g | 2 | 77.77-78.11 | 0 | 77,769,050-78,105,103 | GWAS_SUS_ |
| 2h | 1 | 80.47 | 0 | 80,474,575 | GWAS_SUS_ |
| 2i | 1 | 84.94 | 0 | 84,944,433 | GWAS_SUS_ |
| 2j | 1 | 98.36 | 0 | 98,358,268 | GWAS_SUS_ |
| 2k | 1 | 104.26 | 0 | 104,262,348 | GWAS_SUS_ |
| 2l | 1 | 105.31 | 0 | 105,312,293 | GWAS_SUS_ |
| 2m | 1 | 122.13 | 0 | 122,130,261 | GWAS_SUS_ |
| 2n | 1 | 131.71 | 0 | 131,713,087 | GWAS_SUS_ |
| 2o | 1 | 134.32 | 0 | 134,326,533 | GWAS_SUS_ |
| 2p | 1 | 138.28 | 0 | 138,281,673 | GWAS_SUS_ |
| 2q | 2 | 145.73 | 0 | 145,725,034-145,725,040 | GWAS_SUS_ |
| 3a | 2 | 0.06 | 0 | 56,713-78,787 | GWAS_SUS_ |
| 3b | 2 | 17.20 | 0 | 17,158,485 | GWAS_LN6_ |
| 3c | 1 | 26.70 | 0 | 26,735,617 | GWAS_LN6_ |
| 3d | 1 | 31.80 | 0 | 31,801,477 | GWAS_LN6_ |
| 3e | 1 | 40.53 | 0 | 40,532,407 | GWAS_SUS_ |
| 3f | 1 | 51.69 | 0 | 51,692,766 | GWAS_SUS_ |
| 3g | 1 | 64.10 | 0 | 64,100,571 | GWAS_SUS_ |
| 3h | 1 | 98.60 | 0 | 98,604,457 | GWAS_SUS_ |
| 3i | 1 | 100.01 | 0 | 100,007,898 | GWAS_SUS_ |
| 3j | 1 | 109.77 | 0 | 109,767,150 | GWAS_SUS_ |
| 4a | 1 | 2.91 | 0 | 2,906,622 | GWAS_SUS_ |
| 4b | 1 | 57.44 | 0 | 57,436,170 | GWAS_SUS_ |
| 4c | 2 | 74.03 | 0 | 74,034,868-74,034,876 | GWAS_SUS_ |
| 4d | 1 | 79.86 | 0 | 79,863,830 | GWAS_LN6_ |
| 4e | 30 | 85.21 – 85.54 | 0 | 85,213,417-85,536,717 | GWAS_SUS_ |
| 4f | 1 | 89.94 | 0 | 89,944,632 | GWAS_SUS_ |
| 5a | 1 | 6.72-6.75 | 0 | 6,719,381-6,745,701 | GWAS_SUS_ |
| 5b | 310 | 7.92– 8.23 | 0 |  | GWAS_LN6_ |
| 5c | 639 | 9.98-10.32 | 0 | 9,978,915- 10,322,066 | GWAS_SUS_ |
| 5d | 597 | 12.50-12.74 | 0 | 12,501,106-12,740,275 | GWAS_SUS_ |
| 5e | 5 | 14.31-14.57 | 0 | 14,313,006-14,565,251 | GWAS_SUS_ |
| 5f | 5 | 16.40-16.46 | 0 | 16,403,371-16,455,644 | GWAS_SUS_ |
| 5g | 1 | 23.82 | 1.28 x 10^-10^ | 23,818,852 | GWAS_LN6_ |
| 5h | 1 | 45.41-46.26 | 2.17 x 10^-5^ – 2.47 x 10^-10^ | 45,408,787 | GWAS_RES-JD6_ |
| 6 | 154 | 23.89-24.15 | 0 | 23,894,776- 24,153,317 | GWAS_SUS_ |
| 7a | 1 | 3.13 | 0 | 3,130,370 | GWAS_SUS_ |
| 7b | 1 | 5.14 | 0 | 5,135,194 | GWAS_LN6_ |
| 7c | 1 | 14.69 | 0 | 14,691,478 | GWAS_RES-JD6_ |
| 7d | 1 | 30.53 | 0 | 30,530,826 | GWAS_LN6_ |
| 8 | 2 | 8.76 | 0 | 8,761,005- 8,761,447 | GWAS_SUS_ |
| 9a | 3 | 0.06-0.07 | 0 | 67,277-75,264 | GWAS_SUS_ |
| 9b | 9 | 3.05-3.06 | 0 | 3,049,334- 3,060,803 | GWAS_SUS_ |
| 12a | 1 | 6.56 | 0 | 6,560,369 | GWAS_SUS_ |
| 12b | 1 | 10.98 | 0 | 10,982,017 | GWAS_SUS_ |
| 14a | 1 | 6.56 | 0 | 6,575,071 | GWAS_SUS_ |
| 14b | 1 | 14.95 | 0 | 14,945,524 | GWAS_SUS_ |
| 16 | 90 | 0.06-2.68 | 0 | 61,836- 2,676,040 | GWAS_SUS_ |
| 17a | 4 | 0.2-0.6 | 0 | 194,494-597,924 | GWAS_SUS_ |
| 17b | 1 | 5.0 | 1.28 x 10^-10^ | 5,008,681 | GWAS_LN6_ |
| 18a | 1 | 0.3 | 0 | 226,649 | GWAS_SUS_ |
| 18b | 1 | 9.03 | 0 | 9,026,351 | GWAS_LN6_ |
| 19 | 3 | 0.18-0.43 | 0 | 175,544- 429,914 | GWAS_SUS_ |
| 20a | 3 | 1.76 | 0 | 1,755,989- 1,760,485 | GWAS_SUS_ |
| 20b | 1 | 2.34 | 0 | 2,339,068 | GWAS_SUS_ |
| 20c | 2 | 3.01 | 0 | 3,098,628- 3,098,693 | GWAS_SUS_ |
| 20d | 2 | 9.52-9.99 | 0 | 9,523,153- 9,991,172 | GWAS_SUS_ |
| 20e | 1 | 10.36 | 0 | 10,357,334 | GWAS_SUS_ |
| 21 | 4 | 6.80-6.87 | 0 | 6,796,817- 6,869,903 | GWAS_SUS_ |
| 22a | 17 | 2.34-2.38 | 0 | 2,342,627-2,377,660 | GWAS_SUS_ |
| 22b | 1 | 3.45 | 0 | 3,446,394 | GWAS_SUS_ |
| 22c | 1 | 4.11 | 2.43 x 10^-8^ | 4,110,017 | GWAS_LN6_ |
| 22d | 3 | 4.46 | 0 | 4,459,640-4,459,701 | GWAS_SUS_ |
| 23a | 15 | 0.01-0.23 | 0 | 12,096-225,332 | GWAS_SUS_ |
| 23b | 2 | 1.84 | 0 | 1,844,399-1,844,978 | GWAS_SUS_ |
| 23c | 261 | 2.00-2.22 | 0 | 2,001,823- 2,224,846 | GWAS_SUS_ |
| 23d | 3 | 6.23 | 0 | 6,228,035-6,232,090 | GWAS_LN6_, GWAS_SUS_ |
| 25 | 1 | 1.29 | 0 | 1,287,484 | GWAS_SUS_ |
| 26 | 1 | 0.88 | 0 | 881,806 | GWAS_SUS_ |
| 27a | 178 | 0.002-1.10 | 0 | 1,839-1,101,198 | GWAS_SUS_ |
| 28 | 14 | 0.06-0.2 | 2.17 x 10^-5^ – 0.00 | 57,575-221,247 | GWAS_SUS_ (13), GWAS_RES-JD6_ (1) |
| ^1^GGA = *Gallus gallus* chromosome.  ^2^Number of SNPs = number of SNPs significantly associated to the trait.  ^3^Interval = the chromosome region spanned by the significant SNPs (in base pairs).  ^4^P-value (range) = the P-value of the most significant SNP and the range of P-values across all significant SNPs, when applicable.  ^5^Top SNP location (bp) = position of the most significant SNP on the chromosome.  ^6^GWAS = type of genome wide association analysis applied: LN6: GWAS_LN6,_ 2 line 6 birds (coded as 1) vs. the rest (11 RCS and 2 SUS; coded as 0); GWAS_SUS,_ 2 SUS (coded as 1) vs. the rest (11 RCS and 2line 6 birds; coded as 0); GWAS_RES-JD6,_ 2 line 6 birds and 4 RCS (namely birds from lines J and D) that are resistant (coded as 1) vs. the rest (7 RCS and 2 SUS; coded as 0). | | | | | |

**Supplementary Table S3**: Gene Ontology (GO) terms and Kyoto Encyclopedia of Genes and Genomes (KEGG) pathways significantly enriched (P-value < 0.005) using genes associated with Marek disease.

**Excel File**

^1^GWAS: GWAS_LN6,_ 2 line 6 birds (coded as 1) vs. the rest (11 RCS and 2 SUS; coded as 0); GWAS_SUS,_ 2 SUS (coded as 1) vs. the rest (11 RCS and 2 line 6 birds; coded as 0); GWAS_RES-JD6,_ 2 line 6 birds and 4 RCS (namely birds from lines J and D) that are resistant (coded as 1) vs. the rest (7 RCS and 2 SUS; coded as 0).

^2^Category: KEGG: KEGG pathway; GO_BP: GO biological process; GO_CC: GO cellular component; GO_MF: GO molecular function.

^3^False discovery rate (FDR) correction for multiple testing.

**Supplementary Figures**

| A  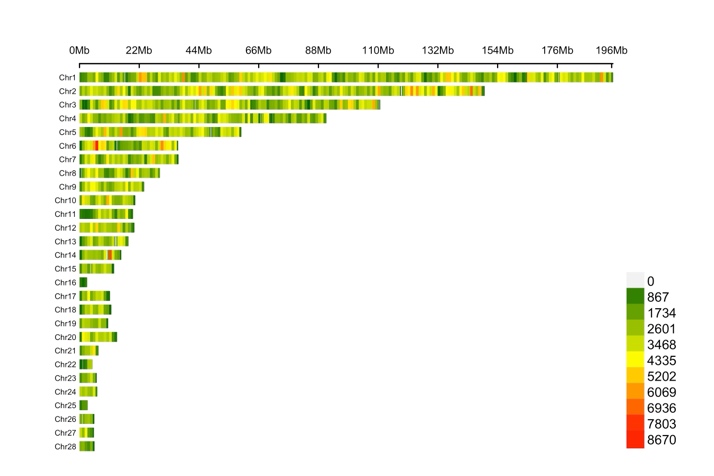  B  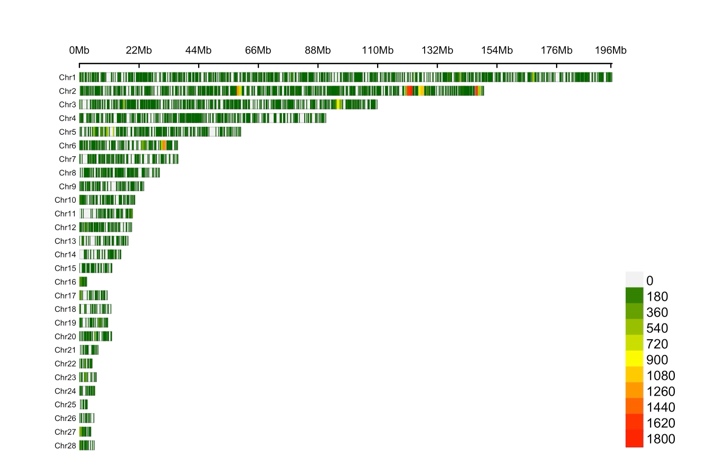 |
| --- |
| **Supplementary Figure S1**: SNP density of a) 2,588,954 common SNPs across all included birds and b) 27,763 segregating SNPs across Gallus gallus autosomes 1-28. |

| A  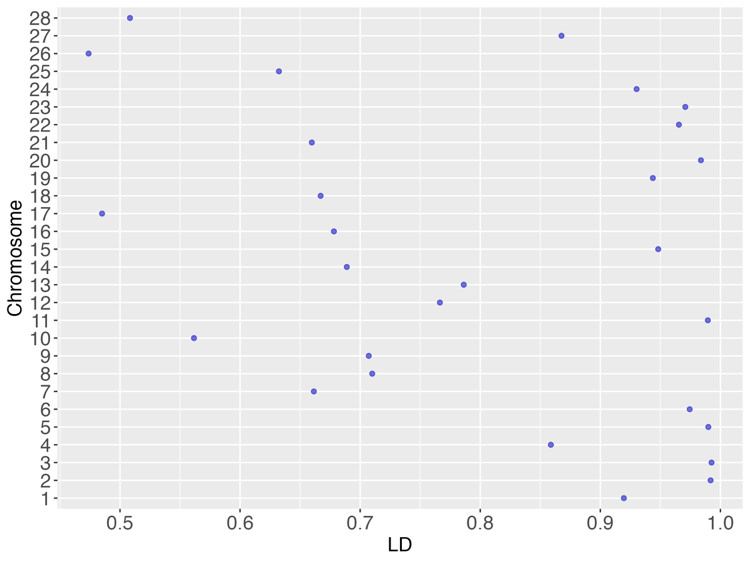 | B  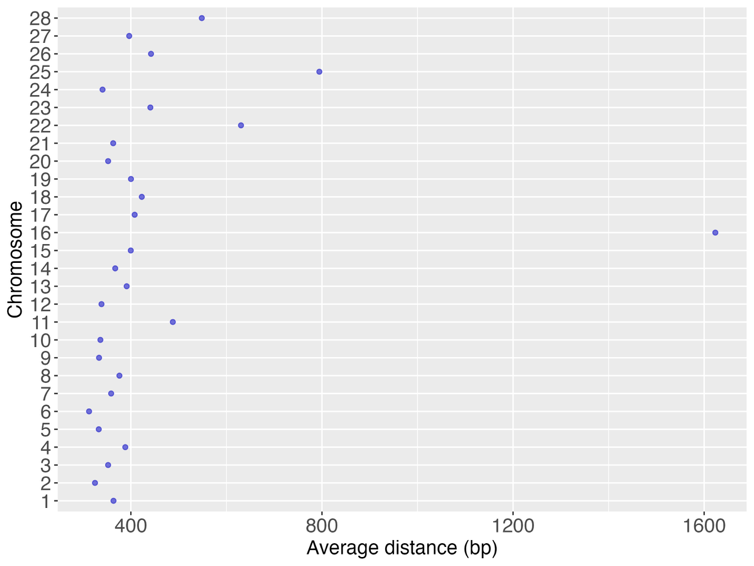 |
| --- | --- |
| **Supplementary Figure S2**: Average Α) linkage disequilibrium (LD) and Β) SNP distance per chromosome (autosomes 1-28). | |

| 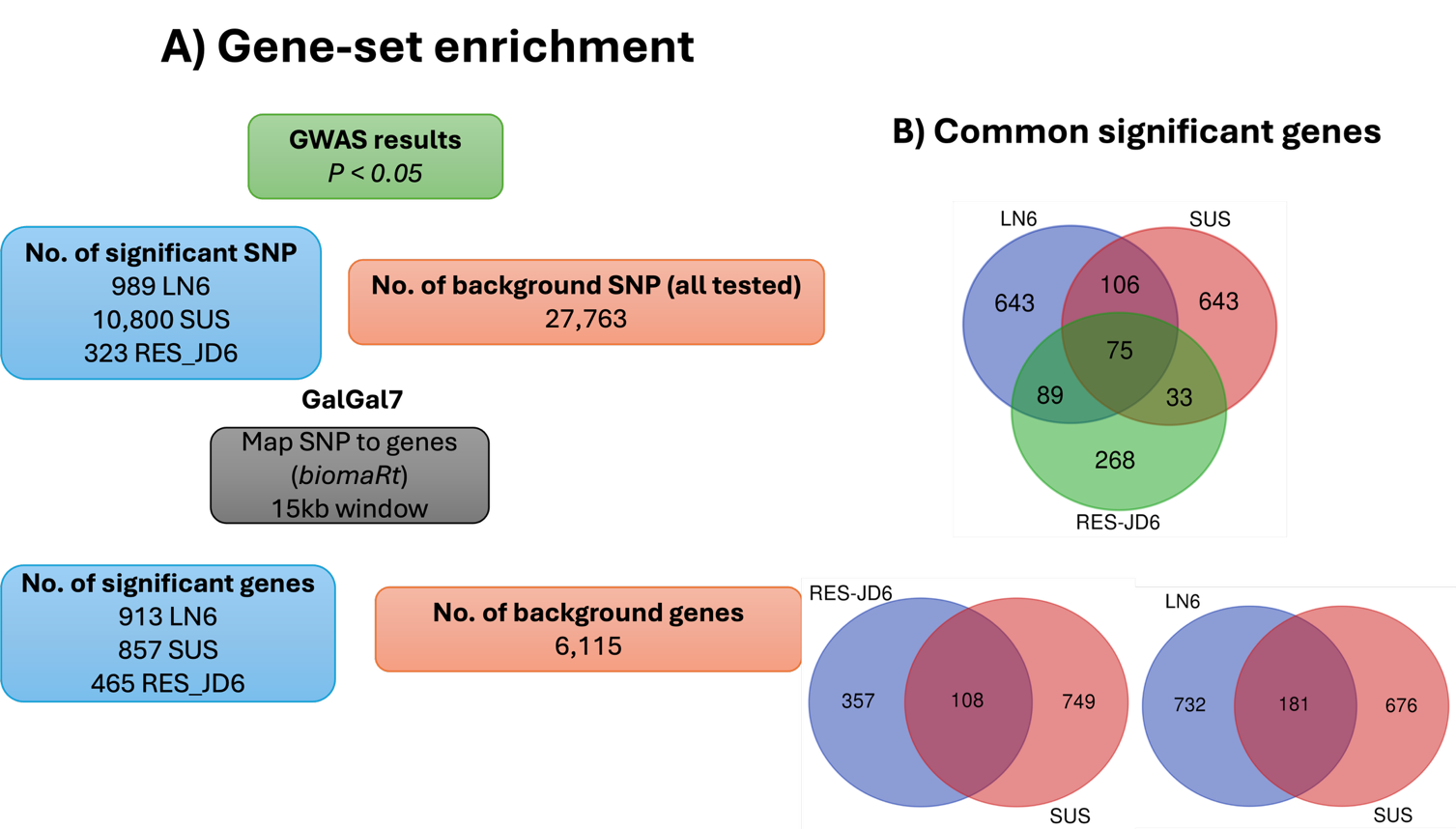  **Supplementary Figure S3:** A) Flowchart for the gene-set enrichment analysis. Significant genes were further analysed in DAVID and IPA; B) Number of common significant genes, among the three genome-wide association analyses (GWAS) conducted.  LN6: GWAS_LN6,_ 2 line 6 birds (coded as 1) vs. the rest (11 RCS and 2 SUS; coded as 0)  SUS: GWAS_SUS,_ 2 SUS (coded as 1) vs. the rest (11 RCS and 2 line 6 birds; coded as 0)  RES-JD6: GWAS_RES-JD6,_ 2 line 6 birds and 4 RCS (namely birds from lines J and D) that are resistant (coded as 1) vs. the rest (7 RCS and 2 SUS; coded as 0).  Venn diagrams were drawn in https://bioinformatics.psb.ugent.be/webtools/Venn/. |
| --- |


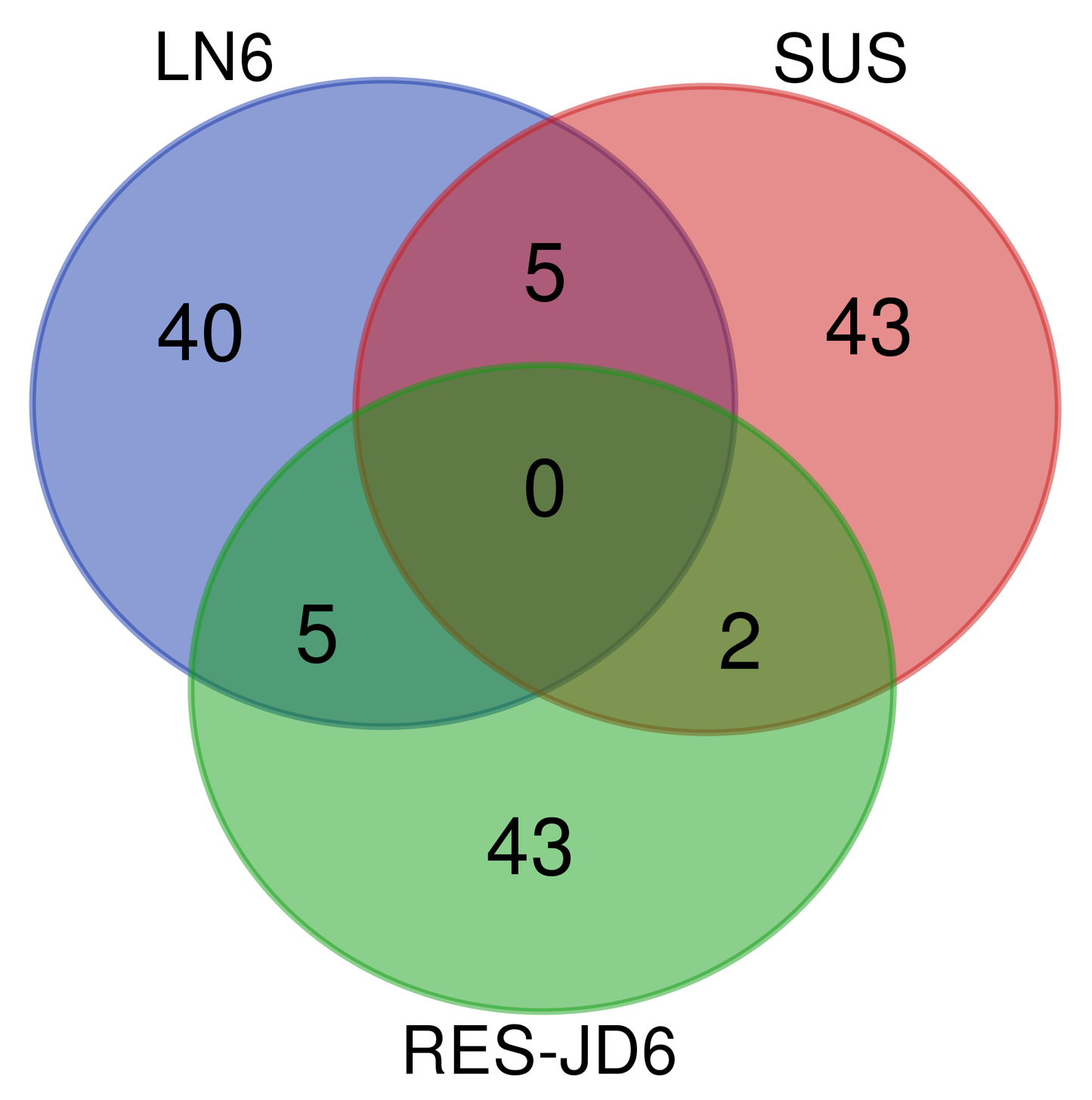


**Supplementary Figure S4**: Top 50 canonical pathways in common among the three genome-wide association analysis (GWAS).

LN6: GWAS_LN6,_ 2 line 6 birds (coded as 1) vs. the rest (11 RCS and 2 SUS; coded as 0)

SUS: GWAS_SUS,_ 2 SUS (coded as 1) vs. the rest (11 RCS and 2 line 6 birds; coded as 0)

RES-JD6: GWAS_RES-JD6,_ 2 line 6 birds and 4 RCS (namely birds from lines J and D) that are resistant (coded as 1) vs. the rest (7 RCS and 2 SUS; coded as 0).

Venn diagrams were drawn in https://bioinformatics.psb.ugent.be/webtools/Venn/.


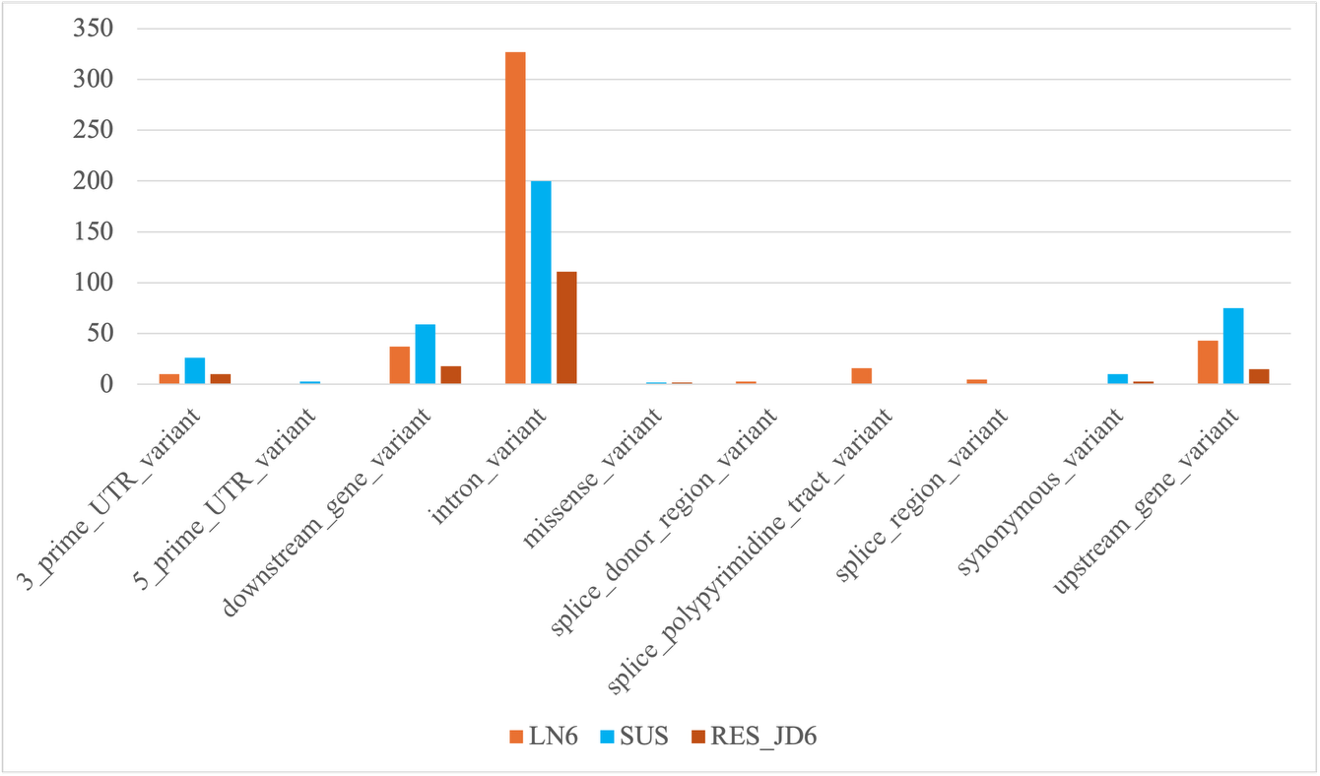


**Supplementary Figure S5**: Variant effect of the significant SNPs of the three genome-wide association studies.

GWAS_LN6,_ 2 line 6 birds (coded as 1) vs. the rest (11 RCS and 2 SUS; coded as 0); GWAS_SUS,_ 2 SUS (coded as 1) vs. the rest (11 RCS and 2 line 6 birds; coded as 0); GWAS_RES-JD6,_ 2 line 6 birds and 4 RCS (namely birds from lines J and D) that are resistant (coded as 1) vs. the rest (7 RCS and 2 SUS; coded as 0).
